# Antimicrobial peptide identification using multi-scale convolutional network

**DOI:** 10.1186/s12859-019-3327-y

**Published:** 2019-12-23

**Authors:** Xin Su, Jing Xu, Yanbin Yin, Xiongwen Quan, Han Zhang

**Affiliations:** 1 College of Artificial Intelligence, Nankai University, Tongyan Road, Tianjin, 300350 China; 2 College of Computer Science, Nankai University, Tongyan Road, Tianjin, 300350 China; 3 Nebraska Food for Health Center, Department of Food Science and Technology, University of Nebraska-Lincoln, 1400 R Street, Lincoln, NE 68588 USA

**Keywords:** Multi-scale convolutional network, Antimicrobial peptide, Deep learning, Fusion model

## Abstract

**Background:**

Antibiotic resistance has become an increasingly serious problem in the past decades. As an alternative choice, antimicrobial peptides (AMPs) have attracted lots of attention. To identify new AMPs, machine learning methods have been commonly used. More recently, some deep learning methods have also been applied to this problem.

**Results:**

In this paper, we designed a deep learning model to identify AMP sequences. We employed the embedding layer and the multi-scale convolutional network in our model. The multi-scale convolutional network, which contains multiple convolutional layers of varying filter lengths, could utilize all latent features captured by the multiple convolutional layers. To further improve the performance, we also incorporated additional information into the designed model and proposed a fusion model. Results showed that our model outperforms the state-of-the-art models on two AMP datasets and the Antimicrobial Peptide Database (APD)3 benchmark dataset. The fusion model also outperforms the state-of-the-art model on an anti-inflammatory peptides (AIPs) dataset at the accuracy.

**Conclusions:**

Multi-scale convolutional network is a novel addition to existing deep neural network (DNN) models. The proposed DNN model and the modified fusion model outperform the state-of-the-art models for new AMP discovery. The source code and data are available at https://github.com/zhanglabNKU/APIN.

## Introduction

In recent years, antimicrobial peptides (AMPs) have attracted lots of attention due to the well-known antibiotic resistance problem. AMPs are polypeptides shorter than 100 amino acids, which are an important part of host defense systems of animals and plants [1]. AMPs have antimicrobial activity under specific circumstances since the difference between microbial and host cells in biochemical and biophysical provides a basis for selective toxicity of AMPs [2]. AMPs exhibit many advantages including fast killing, low toxicity, and broad range of activity [3]. Besides, AMPs show a lower likelihood for antimicrobial resistance compared to many antibiotics [4]. Due to the advantages of AMPs, they have been a popular research area of bioinformatics.

To identify AMPs, many computational tools are proposed such as CAMP [5], CAMPR3 [6], ADAM [7], AMPer [8], AntiBP [9], AntiBP2 [10], AVPpred [11], iAMP-2 L [12], EFC-FCBF [13], classAMP [14] and web-based antimicrobial peptide prediction tools [15]. Many of these tools applied various machine learning methods. For example, support vector machine (SVM), random forest (RF), and artificial neural network (ANN) were employed in CAMP. To apply machine learning methods, feature engineering is a necessary step. The most popular features for AMPs are amino acid composition. For example, AntiBP employed basic amino acid counts over the full peptide as the features. The pseudo-amino acid composition (PseAAC) method is also applied in some methods [16].

For machine learning methods, feature construction of protein sequences relies heavily on domain knowledges. To avoid the complexity of feature engineering and remove the burden of feature construction, many deep learning models have been applied to various problems in bioinformatics [17] such as protein structure prediction [18, 19], protein classification [20], biomedical imaging recognition [21, 22]. To apply deep learning to the problem of AMP identification, a deep neural network (DNN) model was proposed [23]. This model employed a convolutional layer [24] and a recurrent layer, which can capture latent features of protein sequences, so it was shown to outperform the state-of-the-art models in AMP identification. Although this model is great, there is still room for improvement. For example, a long short-term memory (LSTM) layer [25] was employed due to its ability to recognize and forget gap-separated patterns in this model. However, this architecture of DNN model is usually applied in natural language processing (NLP) [26, 27], and is not appropriate for AMP identification in our experiments which is listed in Table 3 for comparison of modified models.

In this paper, we have designed a multi-scale convolutional network which contains multiple convolutional layers of different filter lengths, and proposed a DNN model based on the multi-scale convolutional network to improve the performance of AMP identification. In the proposed model, we have employed an embedding layer and a multi-scale convolutional network. The embedding layer can capture semantic information of amino acids by converting each of them into a numerical vector. The distance between vectors can represent the relation between the corresponding amino acids. Many word embedding models, such as word2vector [28] and gloves [29], are widely used in text recognition tasks. The choice of a multi-scale convolutional network is due to its ability to capture latent features of motifs. Since a multi-scale convolutional network contains multiple convolutional layers, it can make use of all latent features captured by their convolutional layers. Because of the ability of the multi-scale convolutional network to capture multi-scale motifs, the proposed model outperforms the state-of-the-art DNN model [23] in AMP identification. To further improve the performance, we also incorporated additional information into the proposed model and proposed a fusion model.

## Results

### Dataset

We adopt four datasets in this paper. The first dataset we used is made by Veltri et al. (2018) [23], containing 1778 AMPs constructed from the APD vr.3 database [30] and 1778 non-AMPs constructed from UniProt [31]. The dataset is split by Veltri et al. (2018) [23] into a training set, a tuning set and a test set and the number of AMP sequences are 712, 354, and 712 respectively. More detailed information of this dataset can be found in Veltri et al. (2018) [23]. In the rest of the paper, this dataset is named DAMP dataset. The second dataset is taken from AntiBP2 [10], which has 1998 peptide sequences. AMPs have ∼75% overlap with DAMP dataset and non-AMPs have no overlap with it. The third dataset is an anti-inflammatory peptide (AIP) dataset, which is from AIPpred [32]. This dataset contains 1258 AIPs and 1887 non-AIPs in training set, 420 AIPs and 629 non-AIPs in test set. The last dataset is from the paper [15], which is composed of 10,278 sequences. Table 1 summarizes the four datasets.

**Table 1:** Dataset summary

Dataset	DAMP dataset [23]	AntiBP2 dataset	AIP dataset	APD3 dataset [15]
Positive samples	1778	999	1678	1713
Negative samples	1778	999	2516	8565

### Setup and runtime performance

The proposed DNN model is constructed using Keras [33], a Python neural network library, with a CPU-based TensorFlow back-end [34]. The weights in our model of 11 are initialized with the default value of Keras. The optimizer is RMSProp whose learning rate is set to 0.0002, and the loss function is ‘binary_crossentropy’. Besides, the batch size is set to 32. Experiments are conducted on a computer with Intel Xeon E3-1226v3 CPU and the RAM of this computer is 8GB. The training of each epoch takes about 56 s and the prediction of a peptide sequence takes 6 ms on average.

### Model tuning

**Fig. 1:**
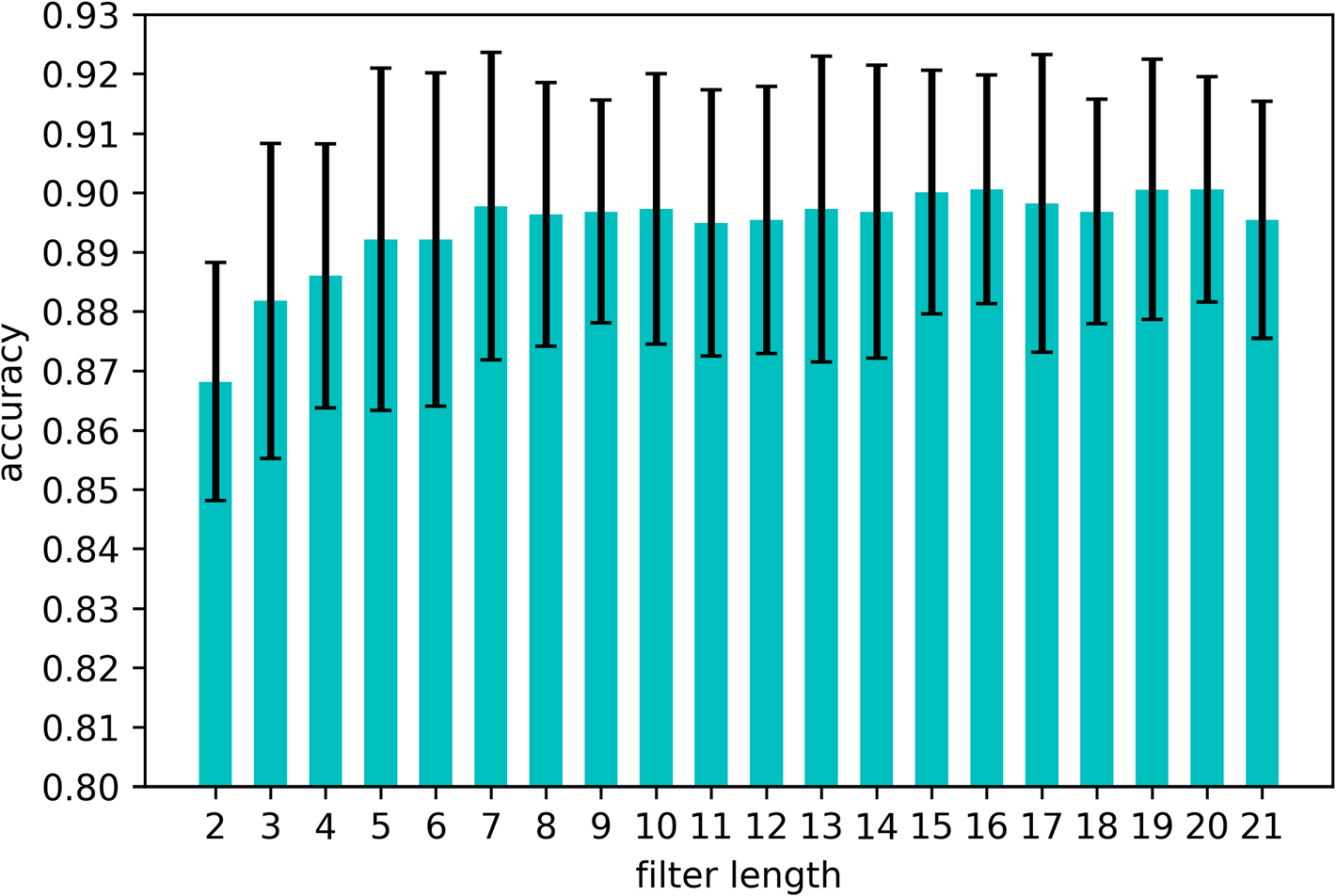
10-fold cross validation performance of the model with single convolutional layer. We replaced the multi-convolutional network with a simple convolutional layer. This figure shows how the modified model performs when the filter length of the convolutional layer changes

First, we want to know how the model performs with only one convolutional layer. We replaced the multi-scale convolutional network with the single convolutional layer. The performance of the modified model with different filter size is shown in Fig. 1. As shown in this figure, the accuracy (ACC) [35] of the modified model is under 89% when this model only contains one convolutional layer whose filter length is short. As the filter length increases, the ACC also increases very fast. The performance of the length between 6 and 20 is similar as shown in Fig. 1. The results of this experiment show that any single convolutional layer whose filter length is shorter than 7 could not capture enough information of a peptide sequence in AMP identification, and the convolutional layers with filter lengths longer than 7 have similar performance in this problem.

**Fig. 2:**
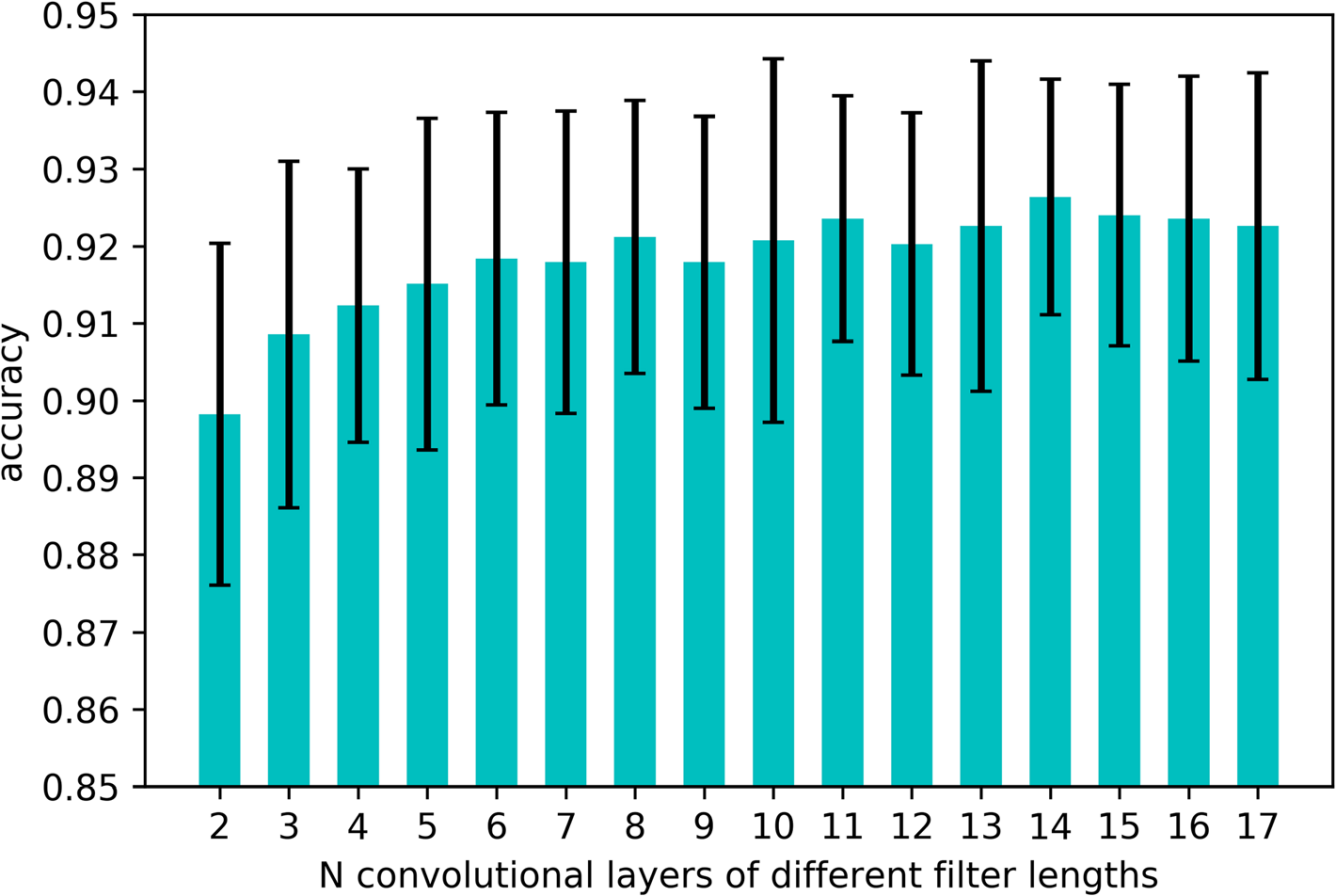
10-fold cross validation performance of the model with different parameter N

Then we want to find the best parameter N in our multi-scale model. Figure 2 shows the performance of the proposed model with different parameter N. As shown in Fig. 2, when N is small, the performance of this multi-scale model is similar to the model with one convolutional layer. Conversely, when N gets larger, the multi-scale model performs better. When *N* = 14, ACC score is the highest with low fluctuation. We finally choose N = 14 in the proposed model.

### Comparison with current main methods

To evaluate the proposed multi-scale DNN model, this model is compared with the state-of-the-art models including the traditional machine learning models and the existing DNN model. Table 2 shows comparison results of the state-of-the-art model. The results show that the proposed model outperforms the existing DNN in all evaluation metrics except sensitivity (SENS). To be specific, the accuracy of the proposed model is about 92.4%, which is 1.3% higher than the existing DNN model, and the specificity (SPEC) is about 94%, which is 1.51% higher than the existing DNN model. Although the highest SENS is achieved by the RF model, the performance of the proposed model is better than the performance of the existing DNN model. The fusion model which makes use of amino acid composition (AAC) [32] and dipeptide composition (DPC) [32] further improves the performance. ACC of the fusion model reaches 92.55%.

**Table 2:** Comparison with the state-of-the-art methods

Method	SENS (%)	SPEC (%)	ACC (%)	MCC	auROC (%)	*P* value
AntiBP2	87.91	90.8	89.37	0.7876	89.36	< 0.001
CAMP-ANN	82.98	85.09	84.04	0.6809	84.06	< 0.001
CAMP-DA	87.08	80.76	83.92	0.6797	89.97	< 0.001
CAMP-RF	92.7	82.44	87.57	0.7554	93.63	< 0.001
CAMP-SVM	88.9	79.92	84.41	0.691	90.63	< 0.001
iAMP-2 L	83.99	85.86	84.9	0.6983	84.9	< 0.001
iAMPpred	89.33	87.22	88.27	0.7656	94.44	< 0.001
gkmSVM	88.34	90.59	89.46	0.7895	94.98	< 0.001
DNN	89.89	92.13	91.01	0.8204	96.48	< 0.001
proposed model	91.01	93.64	92.41	0.8486	97.23	< 0.001
fusion model with DNN	88.48	93.26	90.87	0.8183	96.24	< 0.001
proposed fusion model	89.89	94.96	92.55	0.8523	97.3	< 0.001

### Modification comparison

We modified the propose model and conducted a modification comparison by replacing or removing some components in the proposed model in order to find out the vital elements of the success of the proposed model and discover the best architecture of DNN model in AMP identification.

To be specific, we have tested the models in which we replaced the embedding layer with one-hot encoding, or replaced multi-scale convolutional network with simple convolutional layer or replaced the pooling1 layers with LSTM layers. Besides, we also have tested models without pooling2 layer or with additional fully connected (FC) layers. The results of modification comparison are shown in Table 3. From the results, we find that the multi-convolutional network is the most important part in our model, and the ACC performance of the model without this component drops to 90.44%. Also, the embedding layer is significant in our model. When we run the model without embedding layer, the ACC performance drops to 91.43%. Additionally, using LSTM to replace pooling1 doesn’t improve the performance of AMP identification and increases runtime. This result implies that LSTM is not a good choice for AMP identification in the proposed model. We also tested a model in which we replaced the pooling1 layers with Gated Recurrent Unit (GRU) layers and its accuracy is 91.43%. Because the structure of GRU is similar to LSTM, the result doesn’t change obviously compared to replacing pooling1 layers with LSTM layers. In addition, the results also show that additional fully connected layer or removing pooling2 would not improve the performance.

**Table 3:** Comparison of modified models

Model	SENS (%)	SPEC (%)	ACC (%)	MCC	auROC (%)
Replacing embedding layer	89.61	93.26	91.43	0.8282	96.75
Replacing multi-scale convolutional network	89.75	91.15	90.44	0.8091	96.08
Replacing pooling1 with LSTM	89.75	93.25	91.5	0.8305	96.27
Without pooling2	91.15	92.56	91.85	0.8371	96.3
Additional FC layers	90.31	93.68	91.99	0.8403	97.09
proposed model	91.01	93.64	92.41	0.8486	97.23

We also analyzed the training time of each modified model. The results are shown in Table 4. The results show that replacing the embedding layer or multi-scale convolutional network reduces the training time but the accuracy decreases. Adding LSTM into the proposed model not only increases the training time but also decreases the accuracy. Besides, adding FC layers or removing pooling2 doesn’t apparently affect runtime.

**Table 4:** Training time of modified models

Model	Time for training on each epoch(s)
Replacing embedding layer	13.69
Replacing multi-scale convolutional network	13.95
Replacing pooling1 with LSTM	121.4
Without pooling2	56.06
Additional dense layers	58.45
proposed model	56.36

### Model performance on other datasets

To find out how the proposed model performs on other datasets, we applied our model to AntiBP2 dataset, AIP dataset and the APD3 benchmark dataset from paper [15].

We used 10-fold cross validation test on AntiBP2 dataset to compare the proposed model with state-of-the-art models. Table 5 shows that the proposed DNN also outperforms other state-of-the-art models on AntiBP2 dataset. The accuracy of this dataset is 93.38%.

**Table 5:** Comparison of the state-of-the-art methods on AntiBP2 dataset

Method	ACC (%)	MCC
CAMP-ANN	81.03	0.624
CAMP-DA	84.28	0.69
CAMP-RF	87.09	0.752
CAMP-SVM	86.69	0.739
iAMP-2 L	86.34	0.735
iAMPpred	92.84	0.858
AntiBP2	91.64	0.831
DNN	92.95	0.86
proposed model	93.38	0.862

We compared the proposed model with the existing DNN [23] and the AIPpred model which is state-of-the-art on AIP dataset. The result is shown in Table 6. From this table, we can see that the accuracy of the proposed model on this dataset is 73.02% (0.38% lower than AIPpred). However, the proposed model performs much better than the existing DNN [23]. When using AAC, DPC and some other features, the proposed fusion model achieves a better performance than AIPpred (ACC is 0.44% higher than AIPpred). This experiment implies that the proposed model has a good applicability and could also be applied to problems of other peptide sequence identification.

**Table 6:** Comparison of the state-of-the-art methods on AIP dataset

Model	SENS (%)	SPEC (%)	ACC (%)	MCC	auROC (%)	*P* value
DNN	59.05	73.61	67.78	0.3273	71.12	< 0.001
proposed model	55.24	84.9	73.02	0.4245	76.8	< 0.001
AIPpred	75.8	71.11	73.4	0.46	80.1	< 0.001
fusion model with DNN	51.67	79.81	68.54	0.3285	71.23	< 0.001
proposed fusion model	60	83.15	73.88	0.4459	78.34	< 0.001

We also tested these methods on the APD3 benchmark dataset. The prediction result is shown in Table 7. The performance metrics indicate that our proposed method and proposed fusion method perform better than other methods. Besides, we used DeLong’s test to get differences between our two proposed methods and other methods with the area under receiver-operating curve (auROC) analysis. The result is shown in Table 8. It also shows that our two proposed methods over-perform other methods.

**Table 7:** Comparison of methods on APD3 dataset

Method	SENS (%)	SPEC (%)	PREC (%)	BalACC (%)	ACC (%)	MCC
CAMP-ANN	83.30	83.36	50.04	83.33	83.35	0.5549
CAMP-DA	88.09	81.25	48.44	84.67	82.39	0.5623
CAMP-RF	94.80	83.44	53.39	89.12	85.34	0.6388
CAMP-SVM	90.54	81.63	49.65	86.09	83.12	0.5848
gkmSVM	–	–	–	–	–	–
iAMP-2 L	88.32	86.12	56.00	87.22	86.49	0.6302
iAMPpred	93.46	79.02	47.12	86.24	81.43	0.5742
DNN	96.96	89.62	65.14	93.29	90.84	0.7471
proposed model	97.90	90.90	68.28	94.40	92.07	0.7761
proposed fusion model	98.25	91.00	68.58	94.62	92.21	0.7802

Note: the mark’—’ means that the result is not available. In this experiment, ‘gkmSVM’ method couldn’t be run successfully because the kernel requirement isn’t satisfied

**Table 8:** Comparison of auROC using DeLong’s test on APD3 dataset

Method 1	Method 2	auROC 1	auROC 2	Difference	P value
proposed model	CAMP-DA	0.9892	0.9069	0.0823	< 0.0001
proposed model	CAMP-RF	0.9892	0.9528	0.0365	< 0.0001
proposed model	CAMP-SVM	0.9892	0.9202	0.0690	< 0.0001
proposed model	gkmSVM	0.9892	–	–	NA
proposed model	iAMP-2 L	0.9892	0.8722	0.1170	< 0.0001
proposed model	iAMPpred	0.9892	0.9466	0.0427	< 0.0001
proposed model	DNN	0.9892	0.9802	0.0091	< 0.0001
proposed fusion model	CAMP-DA	0.9918	0.9069	0.0849	< 0.0001
proposed fusion model	CAMP-RF	0.9918	0.9528	0.0391	< 0.0001
proposed fusion model	CAMP-SVM	0.9918	0.9202	0.0716	< 0.0001
proposed fusion model	gkmSVM	0.9918	–	–	NA
proposed fusion model	iAMP-2 L	0.9918	0.8722	0.1196	< 0.0001
proposed fusion model	iAMPpred	0.9918	0.9466	0.0453	< 0.0001
proposed fusion model	DNN	0.9918	0.9802	0.0117	< 0.0001
proposed fusion model	proposed model	0.9918	0.9892	0.0026	< 0.0001

Note: the mark’—’ means that the result is not available. In this experiment, ‘gkmSVM’ method couldn’t be run successfully because the kernel requirement isn’t satisfied

## Discussion

We have designed a multi-scale convolutional DNN model to identify AMP sequences. In terms of accuracy, it overperforms other methods on three datasets. Although the proposed model and the proposed fusion model have no obvious advantage over AIPpred, the former models use less information from sequences and they’re easily to use. The propose model takes a little longer time than some modified model but the runtime is acceptable and the prediction accuracy has significant improvements.

## Conclusion

To identify AMPs, we have proposed a DNN model based on the multi-scale convolutional layers. The proposed DNN model mainly employs the embedding layer and the multi-scale convolutional network. Through the embedding layer, each amino acid in a peptide sequence is converted into an embedding vector. The multi-scale convolutional network can capture the local features, and its max pooling layers and convolutional layers of different filter lengths can help with the feature selection. This model focusing on the local context could improve the performance of AMP identification. Furthermore, we have incorporated additional information into the proposed model and developed a fusion model. Compared with the state-of-the-art models, our proposed model achieved better performance. Through the model modification comparisons, we found that the model without multi-scale convolutional network achieved the worst results, which means the multi-scale convolutional network is the most important part in our model. We also applied the proposed model and proposed fusion model to other datasets including an AMP dataset and an AIP dataset and the APD3 benchmark dataset. The results show that the fusion model could achieve a better performance and our proposed model is applicable for other peptide identification.

## Methods

### Structure of our proposed DNN

First, we tested and analyzed the state-of-the-art DNN model which contains a LSTM layer. The LSTM layer applied to AMP identification focuses on the whole sequence without caring about short motifs. However, it is believed that proteins with similar functions may share some short motifs [32]. This means that we can predict AMPs based on these motifs shared with known AMPs.

**Fig. 3:**
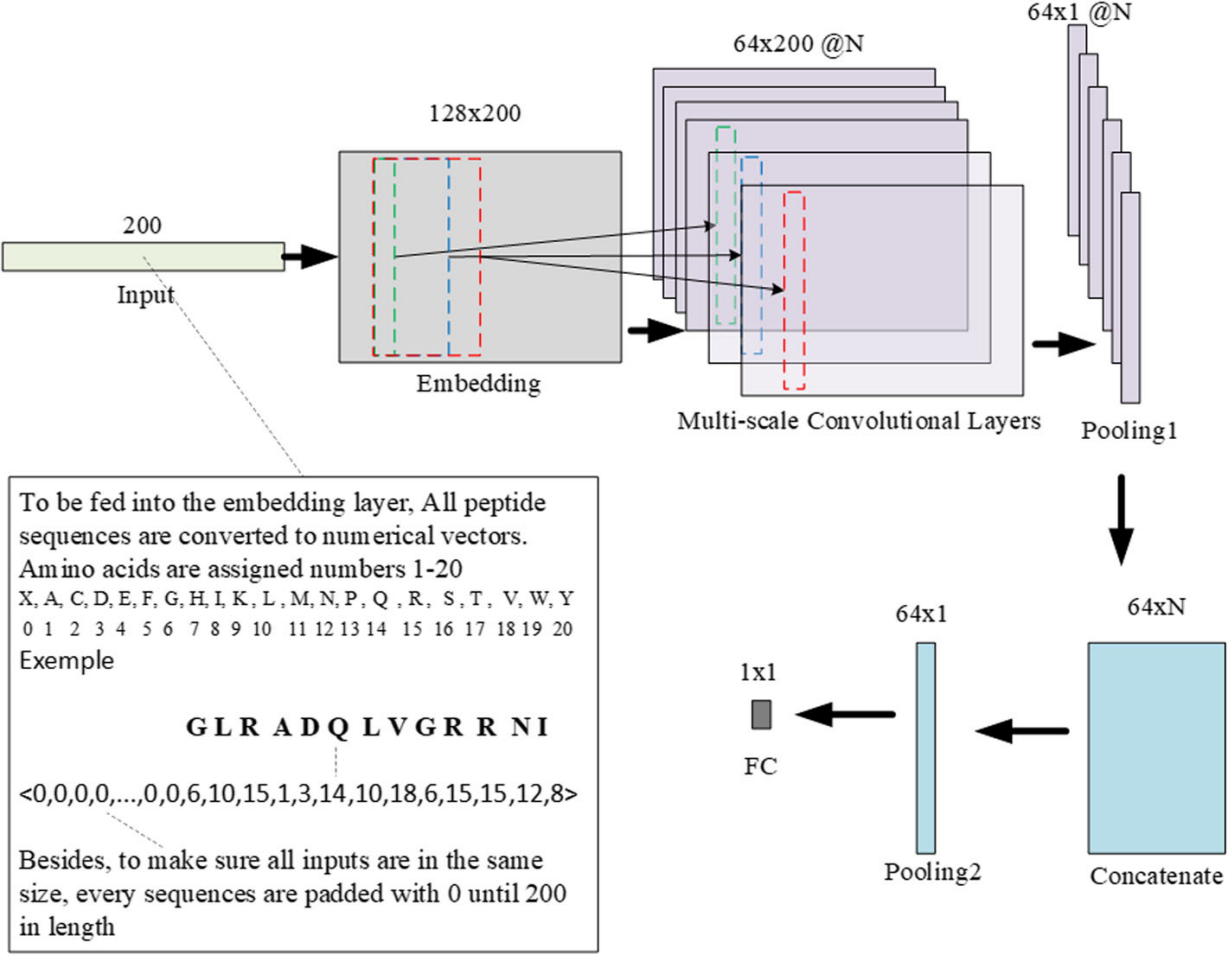
The structure of the proposed model. The proposed model mainly uses embedding layer and convolutional layers. All sequences are encoded into numerical vectors of length 200 and are fed into the embedding layer. Each embedding vector dimension is 128. Then the outputs of embedding layer are fed into N convolutional layers. Each convolutional layer uses 64 filter kernels. These outputs are connected to feed into a max pooling layer and outputs of the pooling layers are concatenated to fed into another max pooling layer. Finally the output will be fed into a fully connection layer and passed through a sigmoid function. The final output is in range [0,1] as the prediction of the input sequence

With this mind, we designed a multi-scale convolutional network, and then proposed a new DNN model based on this network. The proposed DNN model mainly employs a multi-scale convolutional network containing many convolutional layers of different filter lengths. Since each convolutional layer can capture motifs of a fixed length, convolutional layers of different filter lengths can detect motifs of different lengths. The structure of our proposed model is shown in Fig. 3, which shows that the proposed model mainly contains an Embedding module, a Convolutional module, a Pooling module and a Fully Connection module. In the proposed model, we used dropout and set the parameter 0.2 to prevent overfitting.

As shown in Fig. 3, the sequence data has to be converted to be fed into the model. A peptide sequence is converted into a numerical vector of length 200, which is larger than the length of the longest sequence. We assigned an integer within 20 to each one of the 20 basic amino acids. The sequence shorter than 200 will be padded with the number 0 to obtain a fixed vector length 200. The padded 0 s will be ignored by the model during later data processing. Then the encoded data will be fed into the embedding layer that can convert the data with discrete representation into a word vector of a fixed size. That they have a dense representation and can represent an abstract symbol (e.g. a word or an amino acid) with a fixed vector can help reduce dimension. Besides, the distance between two word vectors can represent the relation between two symbols. Compared to the one-hot encoding, the word vector is more compact. As a result, the embedding layer will output a sequence matrix given an amino acid sequence. The matrix has a fixed-dimension of 128 × 200 in our model. The embedding layer will be trained with the whole model.

In the Convolutional module, we employed a multi-scale convolutional network containing N convolutional layers of different filter lengths. A filter will be activated when a matching motif is detected. An amino acid sequence embedding presentation is given as

$$ X=\left[{v}_1,{v}_2,\dots, {v}_{200}\right] $$

where *v*_*i*_(∈*R*^128^) is the embedding vector of *i*-th amino acid. To extract local contexts, the output of each convolutional layer is as

$$ {y}_i^{(f)}=\delta \left({w}^f{x}_i+{b}^{(f)}\right),f=1,2,3,\dots, 64 $$

where δ(∗) means a non-linear activation function which is Rectified Linear Unit (ReLU) [36] in our model, *w*^(*f*)^ and *b*^(*f*)^ are weight and bias of *f*-th filter, and *x*_*i*_ is *i*-th part which is to be convolved. *x*_*i*_ is as [*v*_*i*_, *v*_*i* + 1_, …, *v*_*i* + *l*_] where *l* is the filter length of this convolutional layer. The Convolutional module takes the most important part in recognizing the AMPs by the short motifs which the convolutional layers can detect. A difference between convolutional layers in the multi-scale convolutional network is the filter lengths. Due to the filters of different lengths, each of the convolutional layers screen motifs of its length and then the results of all convolutional layers are different. To be specific, the filter lengths of all N convolutional layers are 2, 4, 6, ..., 2 N.

Each convolutional layer’s output is fed into a max pooling layer. The pooling layer helps reduce over-fitting. Besides, the max pooling is similar as feature selection, which selects the feature with max value. Next, to make use of motifs of different size, all pooling layers’ outputs are concatenated. In other words, the results of all different convolutional layers are concatenated. Then the concatenated layer’s output is fed into another max pooling layer. Finally, the output of pooling layer is fed into a fully connected layer to get the final prediction. The final dense layer uses a sigmoid function and its output is in the range [0,1]. The final output greater than 0.5 means the input sequence is an AMP, otherwise, a non-AMP.

As described above, recurrent neural network (RNN) or LSTM were not used in the proposed model. In our experiments, adding LSTM or RNN did not improve the performance of the proposed model significantly. The results of experiments are discussed in Results section. The features of motifs which convolutional layers detect are used for our identification of new AMPs.

### Model tuning and metrics

We evaluate our proposed model based on sensitivity (SENS), specificity (SPEC), precision (PREC), balanced accuracy (BalACC), accuracy (ACC) [35] and Matthew’s Correlation Coefficient (MCC) [37]. All of them are based on the number of true positive (TP), true negative (TN), false positive (FP), false negative (FN). They are defined as

$$ SENS=\frac{TP}{\left( TP+ FN\right)}\times 100\% $$

$$ SPEC=\frac{TN}{\left( TN+ FP\right)}\times 100\% $$

$$ PREC=\frac{TP}{\left( TP+ FP\right)}\times 100\% $$

$$ BalACC=\frac{1}{2}\times \left(\frac{TP}{\left( TP+ FN\right)}+\frac{TN}{\left( TN+ FP\right)}\right)\times 100\% $$

$$ ACC=\frac{TP+ TN}{\left( TP+ TN+ FP+ FN\right)}\times 100\% $$

$$ MCC=\frac{\left( TP\times TN\right)-\left( FP\times FN\right)}{\sqrt{\left( TP+ FN\right)\times \left( TN+ FP\right)\times \left( TP+ FP\right)\times \left( TN+ FN\right)}} $$

Besides, we also make use of auROC [38]. The receiver operating curve (ROC) can represent the performance of a model by showing the TP rate as a function of FP rate. As the discrimination threshold changes, the TP rate and FP rate change. The auROC is the area under the ROC, which is in range [0.5,1]. 0.5 means random guess, while 1 means that the prediction is always correct.

To reflect different filter lengths bring about different prediction results, a 10-fold cross validation based on a single convolutional layer was conducted. Besides, to find out the best parameter N which is the number of convolutional layers in the multiscale convolutional network, we conducted a 10-fold cross validation to evaluate the parameter N. In this procedure, we merged the training set and tuning set and only took ACC into consideration to choose N. After N was chosen, we merged the training set and tuning set as a new training set to train the proposed model and then evaluated the proposed model and compared it with the state-of-the-art models based on the prediction results of the test set.

### Fusion model

To further improve the performance of the proposed model, redundant information [39] of a peptide sequence is incorporated into the proposed model via a hybrid approach. We combined the proposed model with a fully connected network into a fusion model to capture multi-type features. Besides peptide sequences, amino acid composition (AAC) [32] and dipeptide composition (DPC) [32] are used in this fusion model. AAC is a vector which represents the fractions of 20 amino acid in its peptide sequence. It is defined as

$$ AAC(i)=\frac{number\ of\ amino\ acid(i)}{Length\ of\ the\ peptide},i=1,2,3,\dots, 20 $$

DPC is a vector which represents the ratio of 400 possible dipeptides in a given sequence. It is calculated as

$$ DPC(i)=\frac{\  number\ of\ dipeptide(i)}{Total\ number\ of\  all\  dipeptides},i=1,2,3,\dots, 400 $$

DPC has a fixed length of 400 which represents the 400 possible dipeptides.

**Fig. 4:**
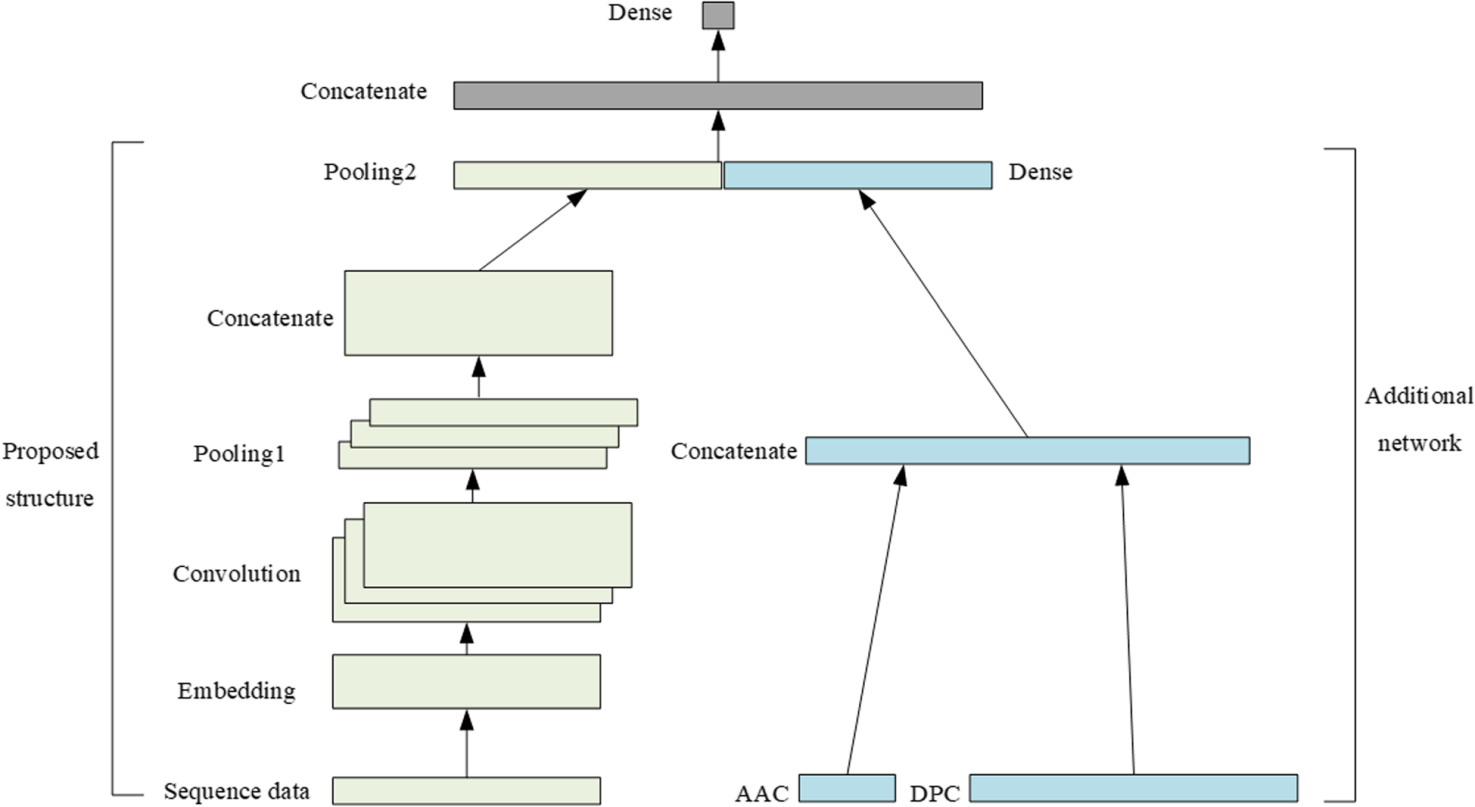
The structure of the proposed fusion model. There are two parts in the fusion model. The proposed structure is on the left. An additional fully connected network is on the right and this part make use of the DPC and AAC of peptide sequences. This network incorporates redundant information into the proposed model

Figure 4 shows the structure of the fusion model. There are two parts in this model. One is the proposed DNN model and another one is an additional fully connected network. The DPC and AAC are concatenated into a vector which has a length of 420. Then this vector is fed into a dense layer with 64 units and each unit use a sigmoid function. The output of this layer with the output of pooling layer in proposed model are concatenated. The concatenated vector is fed into a final dense layer with 1 unit. The final dense layer uses a sigmoid function and its output is in the range [0,1]. We only make use of DPC and AAC in this model, which are easy to obtain, and thus this model also can be applied to any sequence dataset.

## Data Availability

The AMP dataset described in Dataset part could be downloaded from http://www.dveltri.com/ascan/v2/ascan.html. The AntiBP2 dataset could be downloaded from http://crdd.osdd.net/raghava/antibp2/. The AIP dataset could be downloaded from http://www.thegleelab.org/AIPpred/. The APD3 dataset could be downloaded from https://www.ncbi.nlm.nih.gov/pmc/articles/PMC5860510/bin/btx081_supp.zip. The source code is available at https://github.com/zhanglabNKU/APIN.

## Abbreviations

AAC: Amino acid composition
ACC: Accuracy
AIPs: Anti-inflammatory peptides
AMPs: Antimicrobial peptides
ANN: Artificial neural network
APD: The Antimicrobial Peptide Database
auROC: The area under the ROC curve
BalACC: Balanced accuracy
DNN: Deep neural network
DPC: Dipeptide composition
FC: Fully connected
FN: False negative
FP: False positive
GRU: Gated recurrent unit
LSTM: Long short-term memory
MCC: Matthew’s correlation coefficient
NLP: Natural language processing
PseAAC: Pseudo-amino acid composition
ReLU: Rectified linear unit
RF: Random forest
RNN: Recurrent neural network
ROC: Receiver-operating curve
SENS: Sensitivity
SPEC: Specificity
SVM: Support vector machine
TN: True negative
TP: True positive
